# Upregulation of Nox4 induces a pro-survival Nrf2 response in cancer-associated fibroblasts that promotes tumorigenesis and metastasis, in part via Birc5 induction

**DOI:** 10.1186/s13058-022-01548-6

**Published:** 2022-07-14

**Authors:** Shakeel Mir, Briana D. Ormsbee Golden, Brandon J. Griess, Raghupathy Vengoji, Eric Tom, Elizabeth A. Kosmacek, Rebecca E. Oberley-Deegan, Geoffrey A. Talmon, Vimla Band, Melissa LT. Teoh-Fitzgerald

**Affiliations:** 1 Department of Biochemistry and Molecular Biology, Buffett Cancer Center, College of Medicine, University of Nebraska Medical Center, 7005 Durham Research Center, 985870 Nebraska Medical Center, Omaha, NE 68198 USA; 2 Department of Pathology and Microbiology, University of Nebraska Medical Center, Omaha, NE 68198 USA; 3 Department of Genetics, Cell Biology and Anatomy, Buffett Cancer Center, College of Medicine, University of Nebraska Medical Center, Omaha, NE 68198 USA

**Keywords:** Cancer-associated fibroblasts, NADPH Oxidase 4, Pro-oxidant, Autophagy, Adaptive stress response pathway, Nuclear factor-erythroid factor 2-related factor 2, CAF survival, Breast cancer progression and metastasis

## Abstract

**Background:**

A pro-oxidant enzyme, NADPH oxidase 4 (Nox4) has been reported to be a critical downstream effector of TGFβ-induced myofibroblast transformation during fibrosis. While there are a small number of studies suggesting an oncogenic role of Nox4 derived from activated fibroblasts, direct evidence linking this pro-oxidant to the tumor-supporting CAF phenotype and the mechanisms involved are lacking, particularly in breast cancer.

**Methods:**

We targeted Nox4 in breast patient-derived CAFs via siRNA-mediated knockdown or administration of a pharmaceutical inhibitor (GKT137831). We also determine primary tumor growth and metastasis of implanted tumor cells using a stable Nox4-/- syngeneic mouse model. Autophagic flux of CAFs was assessed using a tandem fluorescent-tagged ptfl-LC3 plasmid via confocal microscopy analysis and determination of the expression level of autophagy markers (beclin-1 and LC3B). Nox4 overexpressing CAFs depend on the Nrf2 (nuclear factor-erythroid factor 2-related factor 2) pathway for survival. We then determined the dependency of Nox4-overexpressing CAFs on the Nrf2-mediated adaptive stress response pathway for survival. Furthermore, we investigated the involvement of Birc5 on CAF phenotype (viability and collagen contraction activity) as well as the expression level of CAF markers, FAP and αSMA.

**Conclusions:**

We found that deletion of stroma Nox4 and pharmaceutically targeting its activity with GKT137831 significantly inhibited orthotopic tumor growth and metastasis of implanted E0771 and 4T1 murine mammary carcinoma cell lines in mice. More importantly, we found a significant upregulation of Nox4 expression in CAFs isolated from human breast tumors versus normal mammary fibroblasts (RMFs). Our in situ RNA hybridization analysis for Nox4 transcription on a human breast tumor microarray further support a role of this pro-oxidant in the stroma of breast carcinomas. In addition, we found that Nox4 promotes autophagy in CAFs. Moreover, we found that Nox4 promoted survival of CAFs via activation of Nrf2, a master regulator of oxidative stress response. We have further shown Birc5 is involved as a downstream modulator of Nrf2-mediated pro-survival phenotype. Together these studies indicate a role of redox signaling via the Nox4-Nrf2 pathway in tumorigenesis and metastasis of breast cancer cells by promoting autophagy and survival of CAFs.

**Supplementary Information:**

The online version contains supplementary material available at 10.1186/s13058-022-01548-6.

## Background

The majority of the cancer stroma compartment is comprised of cancer-associate fibroblasts (CAFs) which are a prognostic factor in invasive breast cancers [1–3]. These fibroblasts differ from the normal resident fibroblasts in that they show a persistent activated phenotype that leads to secretion of a variety of pro-tumorigenic cytokines, growth factors and extracellular matrix (ECM) molecules. These stroma cells show great potential as drug targets in breast cancer, given their genomic stability and abundance. Increasing evidence also demonstrates that targeting CAFs could be a promising clinical approach. However, targeting these stromal cells remains a challenge due to their heterogeneity. Therefore, a better characterization of the underlying mechanisms that promote the activated phenotype of CAFs is necessary for targeting these cells with therapy. One such mechanism that we have previously shown to influence the activated fibroblast phenotype is an upregulation of a pro-oxidative enzyme, NADPH oxidase 4 (Nox4) [4].

While mitochondria are considered the major source of cellular ROS generation, significant levels of ROS can be contributed through the activation of Nox enzymes. These membrane-associated enzymes function in generating superoxide (O_2_^•−^) or its derivative, hydrogen peroxide (H_2_O_2_), when triggered by specific stimuli, which include a number of growth factors and cytokines [5]. Depending on their subcellular distribution, the Nox family of proteins execute a wide range of ROS-mediated biological functions. By activating/inactivating oxidative-sensitive kinases or protein tyrosine phosphatases, Nox-derived ROS are now recognized to be important mediators that provide “fine-tuning” of a variety of signaling cascades. Recently, research suggested that Nox activation is linked to the etiology of cancer [6]. Among the seven Nox family members, Nox4 is the only one that is constitutively active in generating ROS (mainly H_2_O_2_ and a small amount of O_2_^−*^) without the need of additional accessory proteins, as are required by the other Nox enzymes [7–9].

To date, not much is known about the role of Nox4 in breast cancer despite a few studies showing that overexpression of this enzyme in cancer cells can promote an aggressive phenotype [10]. In fibroblasts, Nox4 has been shown to be one of the downstream effectors of TGFβ in mediating fibroblast activation during fibrosis in cardiac and pulmonary fibroblasts [11, 12] but the role of Nox4 in CAFs is not fully understood. We have previously shown that Nox4 is essential in mediating the myofibroblast phenotype in RMF-HGF [4]. RMF-HGF is an experimentally-generated mammary CAF model isolated from reduction mammoplasty and transduced with hepatocyte growth factor (HGF) [13]. These pro-tumorigenic fibroblasts showed a significant upregulation of Nox4 as compared to its parental non-malignant fibroblasts (RMF: isolated from reduction mammoplasty). Moreover, inhibition of Nox4 attenuated the collagen contraction ability, myofibroblast marker expression, and pro-invasive properties of RMF-HGF [4], suggesting a potentially important role of Nox4-generated ROS in CAFs.

The nuclear factor erythroid 2-related factor 2 (Nrf2) is a transcription factor that is activated in response to oxidative stress and electrophilic stress. It trans-activates antioxidant genes and detoxification genes to restore cellular redox balance and provides phase II detoxification response in eukaryotes [14]. Under basal conditions, Nrf2 is kept at very low levels where Keap1 functions as an adapter for the E3 ubiquitin ligase Cullin-3 (CUL3) constitutively targets Nrf2 for ubiquitination and degradation. Under oxidative stress, Keap1 is inactivated, leading to a translocation of Nrf2 to the nucleus, where it activates genes that contain an antioxidant response element (ARE) [15]. Nrf2 has dual functions in cancer, where its activation prior to tumor initiation or progression is preventive, but activation of Nrf2 in an established tumor enables increased proliferation and aggressiveness, as well as resistance to therapies [16, 17], making Nrf2 a promising anti-cancer target.

In this study, we have further investigated the influence of Nox4 on fibroblast activation and their tumor-promoting function. We found that stroma deletion of Nox4 and administration of a pharmaceutical Nox4 inhibitor (GKT137831) resulted in suppression of orthotopic mammary tumor growth and metastasis in two syngeneic models, suggesting a prominent role of stroma Nox4 in oncogenesis. To show that this pro-oxidant is clinically relevant, we obtained a panel of patient-derived breast CAFs and found a significant upregulation of Nox4 expression levels in all CAFs compared to the normal mammary fibroblasts (also referred to as RMFs). Furthermore, Oncomine analysis showed that Nox4 is ranked in the top 1–3% of most significantly upregulated genes in breast carcinoma stroma versus normal stroma, whereas only a slight upregulation is seen in breast cancer epithelial cells. In addition, we showed that Nox4-generated ROS provides a pro-survival advantage in CAFs via promoting an autophagic phenotype. We later found that activation of the Nrf2 antioxidant pathway contributes to this pro-survival feature in CAFs. Taken together, our study strongly supports a role of Nox4 in breast CAFs and that this pro-oxidant could be a promising stroma target to interfere with the tumor supporting network in breast cancer.

## Methods

### Cell lines, breast CAFs, growth conditions, and reagents

MDA-MB231 (ATCC, HTB-26, RRID:CVCL_0062) and 4T1 (ATCC, CRL-2539, RRID:CVCL_0125) were cultured as previously described [18]. Authentication was verified by short tandem repeat DNA profiling. Breast CAFs were obtained from Asterand Biosciences (now BioreclamationIVT) and were certified and accrued with strict consensual control, quality assurance and accurate clinical data. All pathology diagnosis were confirmed by board certified pathologists and each specimen is scored for condition, cancer percentage, necrosis and other physical factors. The E0771 mouse breast cell line was obtained from CH3 Biosystems and were cultured as recommended by the supplier. Reduction mammoplasty fibroblasts expressing human HGF (RMF-HGF) and their parental normal fibroblasts (RMF) were generated and gifted by Dr. Charlotte Kuperwasser (Tufts University, Boston, MA, USA) [13]. All cultures were regularly tested for Mycoplasma.

### Antibodies and reagents

Antibodies used were: anti-Nox4 (Abcam Cat# ab133303, RRID:AB_11155321), anti-Nrf2 (Abcam Cat# ab62352, RRID:AB_944418), anti-Keap1 (Proteintech Cat# 10,503–2-AP, RRID:AB_2132625), anti-beta-actin (Cell Signaling Technology Cat# 3700, RRID:AB_2242334), anti-p62 (Cell Signaling Technology Cat# 5114, RRID:AB_10624872), and Phospho-p62 (Ser349) (Cell Signaling Technology Cat# 16,177, RRID:AB_2798758). Secondary horseradish peroxidase conjugated antibody used were anti-rabbit (1:5000, Thermo Fisher Scientific, Cat# G-21234, RRID AB_2536530) and anti-mouse (1:6000, Thermo Fisher Scientific Cat# A24524, RRID:AB_2535993).

Brusatol was purchased from Sigma Aldrich (SML1868) and was prepared in DMSO (5 mg/ml). DMF was purchased from Sigma-Aldrich (242,926) and was dissolved in DMSO (5 mg/ml). The selective Nox4 inhibitor GKT137831 was kindly supplied by Genkyotex (S.A., Geneva, Switzerland).

### Real time PCR primers

SDF1: Forward—5′ GAC CCA ACG TCA AGC ATC TC 3′.

Reverse—5′ CGG GTC AAT GCA CAC TTG TC 3′.

αSMA: Forward—5′ GCG TGG CTA TTC CTT CGT TA 3′.

Reverse 5′ TCA GGC AAC TCG TAA CTC TTC TC 3′.

PDFGRα: Forward—5′ TGC CTG ACA TTG ACC CTG T 3′.

Reverse—5′ CCG TCT CAA TGG CAC TCT CT 3′.

Birc5: Forward—5′ ACCACTTCCAGGGTTTATTCC 3′.

Reverse—5′ CAGGCAGAAGCACCTCTG 3′.

FAP: Forward—5′ TCCAGAATGTTTCGGTCCTG 3′.

Reverse—5′ CTATATGCTCCTGGGTCTTTGG 3′.

IL10: Forward—5′ AGG CTG AGG CTA CGG CGC 3′.

Reverse—5′T TAG ATG CCT TTC TCT TGG AG 3′.

### Macrophage differentiation and polarization

Peripheral blood mononuclear cells (PBMCs) were isolated from human donor whole blood. Primary human monocytes and peripheral blood leukocytes were separated via elutriation by the UNMC Elutriation core. Monocytes, and PBLs were used immediately after separation or were cryopreserved in liquid nitrogen before use. PBMCs and monocytes were maintained at 37˚C in 5% CO_2_ in RPMI media with glutamine, 10% fetal bovine serum, penicillin and streptomycin added. Polarization of macrophages was induced as we previously described (). Specifically, monocytes were differentiated and polarized to M2 macrophages with M-CSF (100 ng/mL, BioLegend #574,806) for 7 days to promote monocyte differentiation and growth. Then, the M-CSF stimulated macrophages were polarized to M2 by addition of IL-4 (20 ng/mL, BioLegend #574,002) for 24 h. 20uM of GKT137831 was added to the monocytes during IL-4 treatment.

### Collagen contraction assay

Activity of CAFs was determined and analyzed by collagen contraction assay as previously described [4].

### Invasion and migration assay

Invasiveness of MDA-MB231 was performed as described [4]. Culture-inserts (Ibidi) were used to measure cell migration. Cell suspension at density of 1 × 10^6^/ml breast cancer cells and 1.6 × 10^5^/ml of fibroblast (70 μL volume) was applied to each chamber of the cell culture insert. The cell culture insert was removed after 16 h leaving a defined cell-free gap of 500 μm. The cells were then treated with brusatol (40 nM) for 24 or 48 h. The cells were fixed and stained with 2% crystal violet. Images were captured every 6 h using an inverted phase contrast microscope. Images were taken at 10X magnification, and the cell-free space was analyzed by using the ImageJ software (RRID:SCR_003070). The percent of wound closure in five randomly chosen fields was calculated.

### Western blot analysis

Cell lysates preparation and Western blot analysis was performed as previously described [19].

### RT–PCR

Total RNA isolation, and reverse transcription, and RT-PCR analysis were performed as previously described [4]. Primer sequences are listed in supplemental Materials and Methods, except for Nox4, which have been previously described [4].

### ROS Detection and glutathione assay

Extracellular H_2_O_2_ levels were measured using the Amplex Red assay as previously described [20]. Glutathione levels were measured by the GSH/GSSG-Glo assay (Promega #V6611) as previously described [20].

### In Situ RNA hybridization for tissue labeling

In situ detection of Nox4 mRNA transcription was performed on the breast cancer tissue microarray (TMA-BR8013) using a RNAscope kit (Advanced Cell Diagnostics, Hayward, CA, USA) with verified probes (Probe-Hs-NOX4) and RNAscope FFPE Reagent Kit, 2.0 HD-Brown Detection Kit, according to the manufacturer's protocol. Specificity of the probe was verified using a positive and a negative control, as provided by the supplier. Housekeeping gene Peptidylpropyl isomerase B (PPIB) was used as an internal-control. Tissues were blindly scored by a board-certified pathologist. Positive staining was determined by brown punctate dots in the nucleus and/or cytoplasm, as recommended by the supplier.

### Immunofluorescent autophagic flux assay

Fibroblasts (normal or cancer associated) were plated on coverslips and transfected with LC3 tandem expressing GFP-RFP using Fugene 6 according to the manufacturer’s protocol. After 2 days, steady-state cells were fixed with 4% paraformaldehyde and Vectashield containing DAPI. Respective filters were used to image red and green LC-3II puncta. All confocal images were captured using Zeiss 710 Confocal Laser Scanning Microscope and analyzed using Zeiss Zen software.

### siRNA transfection

All siRNA transfections were performed as previously described [4]. The siCon (#4,390,843) and the siNox4 (ID#s224159) were purchased from Thermo Fisher Scientific, Waltham, MA, USA. A positive control and a negative control were used to verify the specificity of the siRNAs, by real time-PCR and western blot analysis.

### Viability assay

10,000 cells/well were seeded in triplicate in 96-well plates (Corning, USA) and allowed to attach for 18 h prior to drug treatments. On the day of assay, 10 μl of the Presto Blue ™ Cell Viability Reagent was added to each well and incubated for 30 min at 37 °C. The signal of each well was detected at 540 nm using Tecan-M200 plate reader.

### Generation of Nox4 inducible fibroblasts

RMF was constructed to overexpress Nox4 under doxycycline induction via the Lentiviral Tet-On 3G inducible expression systems (Takara Bio USA, Inc.).

### In vivo tumor study

Balb/c mice and C57BL/6 J mice (wild type and Nox4^−/−^) at 8–10 weeks old were purchased from The Jackson Laboratory (Bar Harbor, ME). Only female mice were used. The animal protocol was reviewed and approved by the Institutional Animal Care and Use Committee of the University of Nebraska Medical Center. The number of animals used per group was determined in consultation with a biostatistician, based on Power calculation to ensure that the desired 80% power is achievable. Orthotopic tumor implantation was performed as previously described [18]. After tumors were palpable, mice were randomly grouped prior to drug administration. In the treatment group, mice were given a daily dose of GKT137831 at 60 mg/kg in 200 uL of vehicle via oral gavage starting 1 week post-cell injection. Control group received the same daily volume of vehicle. Tumor volume was measured with a caliper within a week after the treatment started.

### Immunofluorescent analysis

Fixed tumor tissue that also contained stroma of the mammary fat pad and adjacent skin were paraffin-embedded and sectioned by the Tissue Science Facility at the University of Nebraska Medical Center. Tissues were de-paraffinized in xylenes and rehydrated through graded alcohols. For antigen retrieval, slides were boiled for 20 min in 10 mM EDTA (pH 9.0). Slides were then allowed to cool for 10 min. Tissues were blocked in 10% horse serum in PBS for 3 h. Following blocking, tissue sections were incubated with a primary antibody for alpha smooth muscle actin (Novus Biological, NB300-978, 1:200) overnight at 4 °C in a humidified chamber. The following day, slides were washed in PBS and stained with a secondary antibody conjugated to AlexaFluor555 (1:250, donkey anti-goat, Invitrogen, cat. A21432). Slides were mounted under coverslips with ProLong™ Gold Antifade with DAPI (Invitrogen, cat. P36931). Slides were imaged using a Leica DM 4000B LED fluorescent microscope, followed by analysis with ImageJ. For the primary tumor region, areas of surrounding stroma were imaged, capturing between 6–10 fields of view. The stromal regions were manually traced to eliminate any tumor tissue or dead space and the number of positive alpha smooth muscle actin cells were normalized to the area analyzed. For lung sections, the sites of tumor metastases were specifically imaged, 7 fields of view were analyzed per lung. Dead space and blood vessels were removed from the analysis. Mean fluorescent intensity per unit area was determined for each field of view.

### Statistical analysis

Statistical analyses were performed using GraphPad Prism 7 software version 7.03 (RRID:SCR_002798). For some experiments, a single-factor ANOVA followed by post hoc Tukey test was used to determine statistical differences between means. Statistical analyses were assessed using a two-tailed Student’s t test. Results shown are representative of at least three separate experiments each performed in triplicate.

## Results

### Inhibiting Nox4 in RMF-HGF reduces their activated myofibroblasts phenotype

**Fig. 1:**
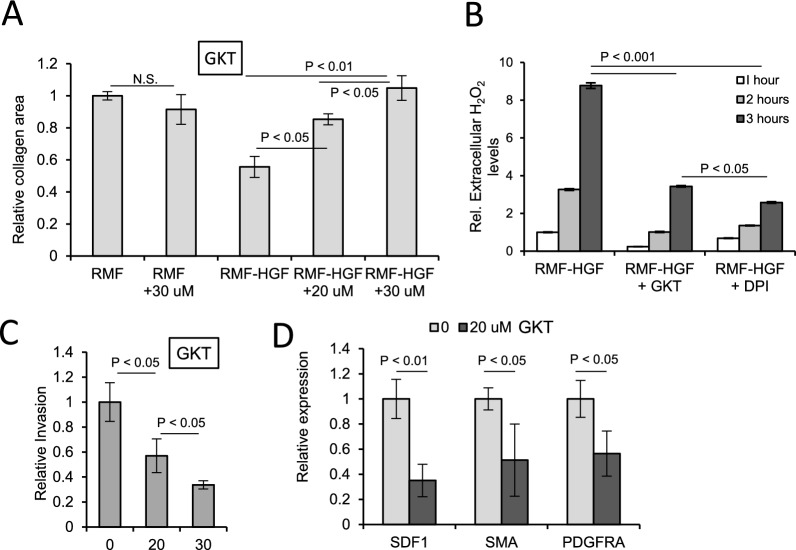
GKT137831 suppressed the activated phenotype of mammary fibroblasts. (**A**) Fibroblasts were treated with GKT137831 at indicated doses for 48 h prior to reseeding in collagen matrix. Area of the collagen discs were measured after 16 h of contraction. Representative results of 3 independent contraction studies are shown here. Error bars = standard deviation of *N* = 3 independent studies. (**B**) GKT137831 effectively reduced cellular oxidative stress, as demonstrated by the levels of H_2_O_2_ released into the cell culture media via AmplexRed assay. DPI was used as a pan-Nox inhibitor. Error bars = standard deviation of *N* = 3 independent studies. (**C**) RMF-HGF that was pre-treated with GKT137831 for 24 h was seeded in a Matrigel invasion lower-well as a chemoattractant and invasion of MDA-MB231 cells was determined after 16 h of seeding. Error bars = standard deviation of *N* = 6 independent experiments. (**D**) Real-time PCR analysis showing mRNA expression of some key myofibroblast markers in GKT137831 treated RMF-HGF

We have previously shown that downregulation of Nox4 via a siRNA approach significantly suppressed the activated phenotype of RMF-HGF [4]. To further show that Nox4 is a promising target in breast CAFs, we evaluated therapeutic efficacies of a pharmaceutical Nox4 inhibitor in this study. We first determined whether the pharmaceutical Nox4 inhibitor, GKT137831, has an effect on the activated phenotype of HGF-overexpressing RMF. Figure 1 A shows that there is a reduction in collagen contractile ability when RMF-HGF were treated with this Nox4 inhibitor, in a dose-dependent manner. Meanwhile, the parental normal mammary fibroblasts, RMF, were resistant to the effect of GKT137831 and showed similar collagen contraction ability in the presence of up to 30 µM of the compound. We have further verified that this Nox4 inhibitor is effective in attenuating Nox4-generated reactive oxygen species (ROS). Figure 1B shows the relative levels of H_2_O_2_ released by RMF-HGF into the extracellular space or culture media over 1 to 3 h. GKT137831 treatment resulted in a more than 50% reduction in the amount of the ROS accumulated in the media at the 3-h time point. The pan-Nox inhibitor, DPI, decreased the levels of extracellular H_2_O_2_ to a similar level as seen with GKT137831, suggesting that Nox4 is the major generator of this ROS in this CAF-like fibroblasts. Next, we tested the effects of GKT137831 on the ability of RMF-HGF to promote invasion of breast cancer cells. Figure 1C shows that the chemotactic ability of RMF-HGF seeded in the lower wells of the Matrigel invasion chamber were significantly suppressed in the presence of the Nox4 inhibitor. We further show in Fig. 1D that the attenuated activated phenotype of the RMF-HGF is in part due to the suppressive effects of GKT137831 on the expression of myofibroblast markers, such as, stroma derived factor 1 (SDF1), alpha smooth muscle actin (αSMA), and platelet derived growth factor receptor alpha (PDGFRα).

### Targeting stroma Nox4-inhibited tumor growth and metastasis

**Fig. 2:**
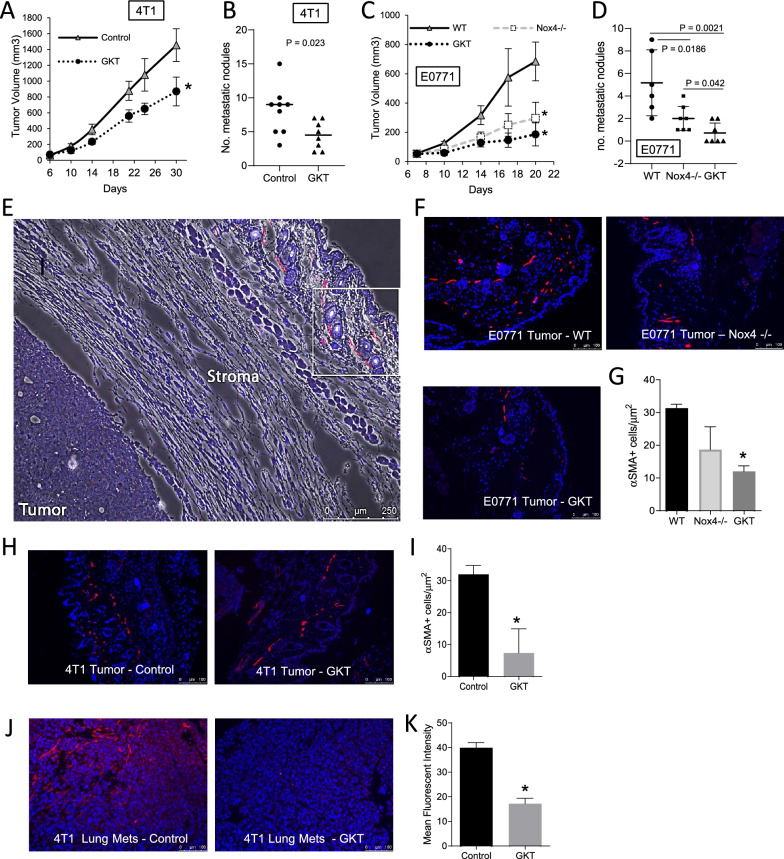
Targeting Nox4 inhibited tumor growth and metastasis in animal models. (**A**) Primary tumor growth induced by murine mammary carcinoma cell line, 4T1 when implanted orthotopically into Balb/c mice. Mice were injected with 2.5 × 10^4^ 4T1 cells into 4th mammary gland. When tumors were palpable on day 6, animals were given daily doses of control vehicle or GKT137831 (60 mg/kg body weight) by oral gavage. * represents *p* < 0.0001 versus control on day 30. (**B**) After 30 days post-tumor cell implantation, mice were euthanized and lungs were harvested, fixed and embedded for metastasis analysis. Lung micro-metastasis was scored based on H&E stained sections. *N* = 9 per group. (**C**) Primary tumor growth of E0771 murine mammary carcinoma cell line in syngeneic C57BL/6 mice, both wild type (WT) and Nox4 knock-out models (Nox4^−/−^). Mice were orthotopically injected with 2 × 10^5^ E0771 cells. One group of animals were given daily doses of GKT137831 (60 mg/kg body weight) by oral gavage starting on day 7. * represents p < 0.0005 versus control or wild type group. Mice were euthanized on day 20 and macro-metastasis (including lungs, lymph nodes, and peritoneal metastatic nodules) was scored as shown in (**D**). *N* = 6–7 per group. Representatives of two independent animal studies were shown here. (**E**) Representative immunofluorescent image (merged with the corresponding bright-field image) of an E0771 induced primary tumor section showing αSMA-positive cells in the stroma region as compared to the tumor area. Random fields of tumor stroma area as indicated by the boxed area are analyzed and quantified for αSMA positivity. (**F**) Representative immunofluorescent images of E0771 tumors post-GKT treatment and Nox4 deletion. (**G**) The number of positive αSMA cells in stroma were quantified for 6–10 fields of view. (**H**) Representative immunofluorescent labeling of α SMA of 4T1 tumors post-GKT treatment. (**I**) The number of positive αSMA cells in stroma was quantified for 6–10 fields of view. (**J**) Representative immunofluorescent labeling of αSMA of 4T1-induced metastatic nodules in lungs. (**K**) Fluorescent intensities of αSMA staining in metastatic sites were quantified by ImageJ. (**G**, **I**, **K**) * represents *P* < 0.05 vs WT. Error bars are standard deviation of *N* = 3

We next determined the efficacy of GKT137831 in suppressing the tumor growth and lung metastasis of the 4T1 syngeneic model. Figure 2A shows that inhibiting Nox4 decreased the 4T1 mammary tumor in Balb/c mice by ~ 40%. Moreover, GKT137831 also significantly reduced lung metastasis in these animals (Fig. 2B). To further demonstrate the biological relevance of stroma-derived Nox4 in breast cancer tumorigenesis and metastasis, we determined the tumorigenicity of E0771, a murine mammary tumor cell line, when orthotopically implanted into either the wild type C57BL/6 mice (Nox4^+/+^) or the KO mice with a constitutive deletion of Nox4 (Nox4^−/−^). Figure 2C shows that Nox4^−/−^ mice had a ~ 56% reduction in the tumor volume on day 17 post-implantation as compared to the wild type group. Stroma deletion of Nox4 also resulted in a significant decrease in metastasis (peritoneal, lymph nodes, and lungs combined) as revealed in Fig. 2D. This suggests that stroma derived Nox4 contributes to tumor progression and metastasis. We then evaluated the efficacies of the Nox4 inhibitor, GKT137831, on the E0771 model in the Nox4^+/+^ background. Administration of this inhibitor resulted in a similar extend of tumor growth suppression, as was observed in the KO group (Fig. 2C). The GKT137831 treated group had significantly reduced metastasis as compared to WT or NOX4^−/−^ mice, where 4/7 GKT137831 treated mice had no signs of metastasis at the endpoint of this study (Fig. 2D). Although this compound is a potent inhibitor of Nox4, it has also been known to target Nox1 (Ki of 140 nM and 110 nM, respectively). The difference seen with GKT137831 treatment and the Nox4 deletion on metastasis is likely due to the dual effect of the inhibitor. NOX1 is well recognized in its role in promoting angiogenesis, making the inhibition of both the NOX1 and NOX4 enzymes by GKT137831, an attractive therapeutic option for cancers.

To further show the influence of Nox4 targeting on stroma phenotype, we assessed the levels of αSMA, as a marker for activated fibroblasts, in FFPE tumors by immunofluorescence labeling. Figure 2E shows a representative image of E0771-induced tumors with αSMA labeled primarily in the stroma of the tumor. Random areas of the stroma (as outlined in the white box of Fig. 2E) were analyzed for αSMA positivity (Fig. 2F). A quantitation analysis reveals that GKT137831 treatment significantly reduced numbers of αSMA positive cells as compared to the WT group, which is in agreement with the tumor inhibition result shown in Fig. 2C. Similarly, we observed a significant decrease in the number of activated fibroblasts in the GKT137831 treated 4T1-tumor stroma (Fig. 2H and I). Furthermore, we have shown that inhibition of Nox4 resulted in a reduction of αSMA intensities in metastatic lung tissues (Fig. 2J and K).

### Oncomine analysis of Nox4 expression in the stroma of breast carcinomas

**Fig. 3:**
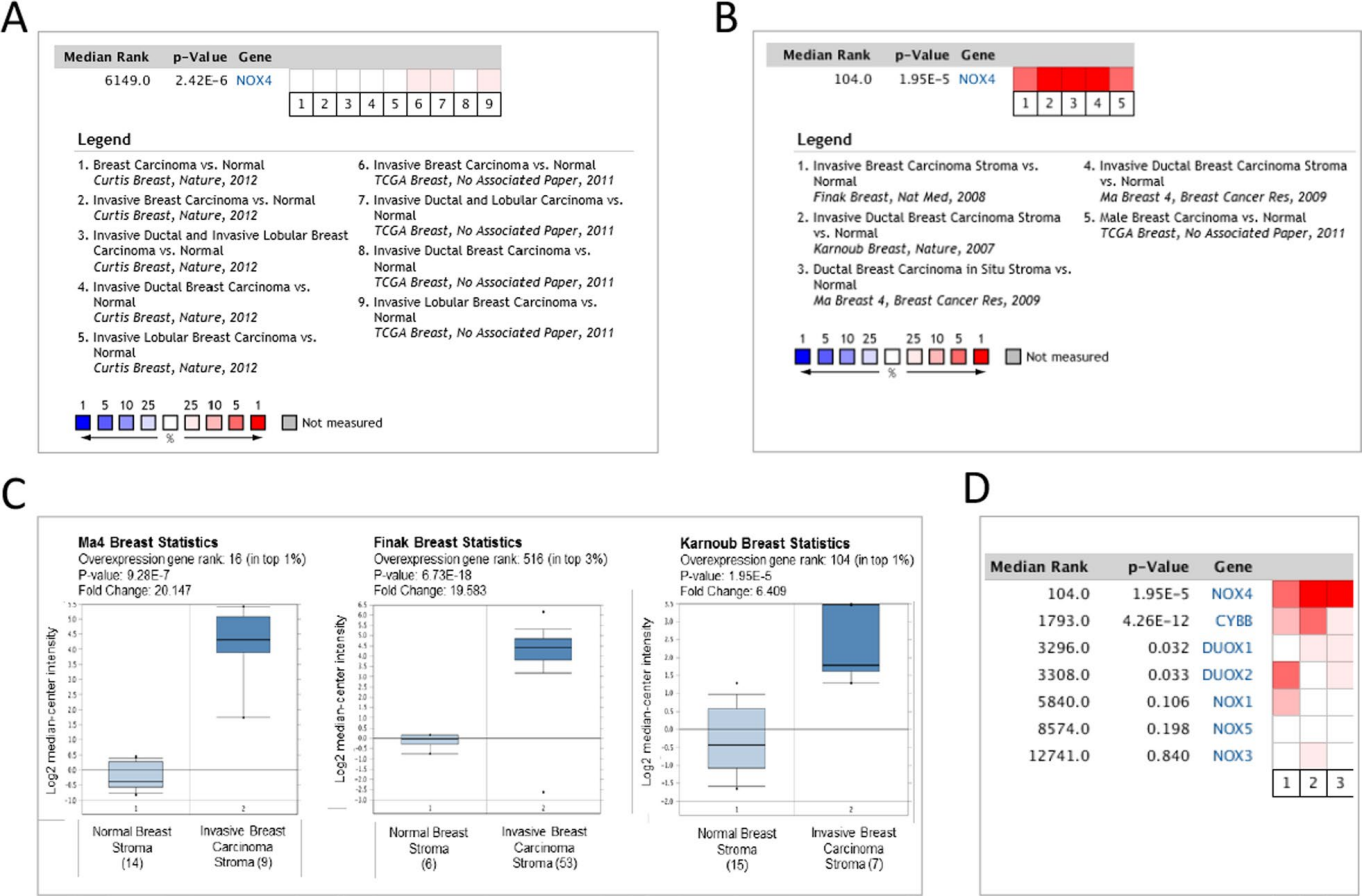
Oncomine *Nox4* analysis in breast cancer. The heatmap represents the relative expression in patients with the indicated breast carcinomas compared with normal tissue. Red box indicates overexpression, while white box indicates insignificant change. The reported median rank and P value consider all indicated studies simultaneously. (**A**) Comparison of Nox4 expression across 9 breast cancer epithelial analyses. (**B**) Comparison of Nox4 expression in 5 subsets of breast cancer stroma analyses. (**C**) Box plots derived from Nox4-gene expression data comparing stroma of invasive breast carcinomas to normal breast stroma from 3 breast stroma studies. The number of samples in each group is indicated in brackets. (**D**) Heat map and median rank comparison of significant differential expression (breast cancer stroma versus normal breast stroma) of all 7 Nox isoforms in the following studies: 1—Ma4 Breast, 2—Finak Breast, and 3—Karnoub Breast

We then performed a microarray database analysis to query the expression levels of Nox4 in breast carcinomas and their stroma vs. normal stromal breast tissues. Figure 3A shows that Nox4 is only slightly upregulated in a small subset of breast carcinomas (median gene rank = 6149). In contrast, Nox4 is ranked among the top 1–3% of the most significantly upregulated genes in the stroma of invasive breast carcinomas versus normal breast stroma (median gene rank = 60, Fig. 3B). Box plots show the degree of change in Nox4 expression as high as 20-fold (Fig. 3C) in the 3 breast stroma studies. Moreover, in comparison to all the Nox enzymes, Nox4 is up-regulated most extensively in breast cancer stroma across all stroma studies (Fig. 3D). Of note, we have also found that targeting Nox4 in macrophages, a major component of immune cells in the stroma, did not alter the pro-tumorigenic M2 macrophage polarization or phenotype (Additional file 1: Fig. S1), further suggesting that CAFs are the main source of Nox4 in the tumor stroma. We have previously shown that sequestering ROS with an antioxidant (manganese porphyrin, MnTE) significantly suppressed M2 macrophage phenotype and that expression levels of Nox1 and Nox4 are not significantly different in M1 vs M2 macrophages (20), implying that Nox4 is not a contributing source of ROS that promotes M2 or tumor-associated macrophage function.

### Upregulation of Nox4 in primary breast CAFs

**Fig. 4:**
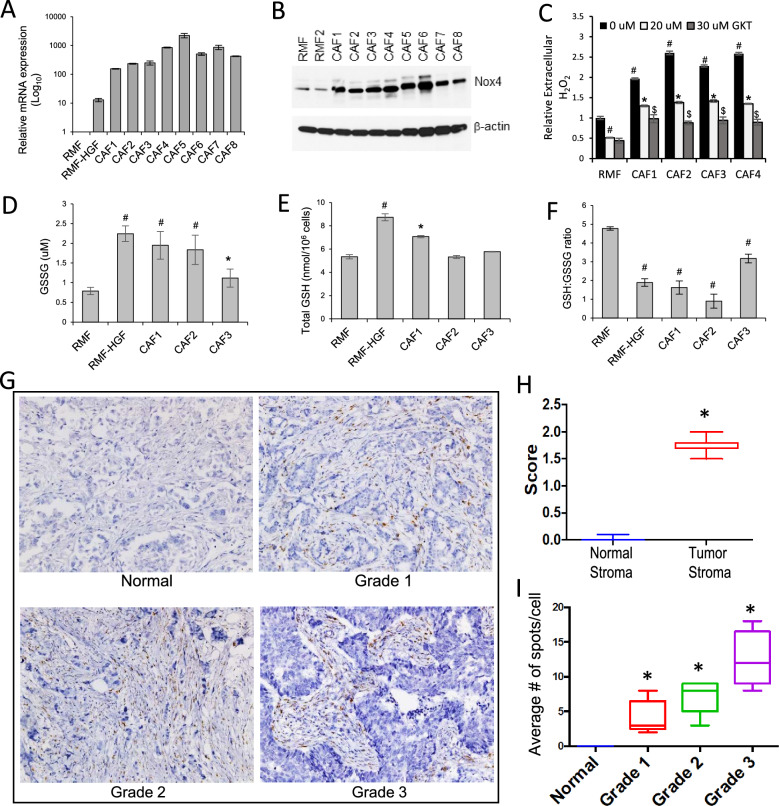
Real-time PCR analysis showing significantly higher Nox4 mRNA expression levels in breast CAFs, including the RMF-HGF, when compared to the normal mammary fibroblasts, RMF. (**B**) Western blot analysis of endogenous Nox4 expression in breast CAFs versus normal mammary fibroblasts. Representative of at least 3 analyses is shown. (**C**) AmplexRed assay indicating the levels of extracellular H_2_O_2_ in CAFs versus RMF after 4 h of incubation with the reagent. Fibroblasts were treated with DMSO control or GKT137831 (20 and 30 uM) for 24 h prior to the assay. Representative data from *N* = 3 independent experiments. Error bars are standard deviations of means. # represents *P* < 0.005 vs. RMF. * represents P < 0.001 in GKT treatment vs. DMSO in individual fibroblasts. $ represents P < 0.001 vs. DMSO of the corresponding CAFs. (**D**-**F**) Cellular glutathione analysis. RMF-HGF and CAFs show higher levels of oxidized glutathione, GSSG when compared to RMF (**D**), resulting in significantly lowered GSH:GSSH ratios (**F**). All bar graph data are mean ± SD of 3 separate samples. *N* = 3 independent experiments. * represents *P* < 0.05, # represents *P* < 0.005 versus RMF. (**G**) In situ detection of Nox4 mRNA transcription on a human breast cancer tissue microarray (TMA-BR8013) using RNAscope kit as described in Methods and Materials. Brown staining was mostly detected in the stroma region of tissues from breast carcinomas. Representative images are shown for each tumor grade. (**H**) Boxplot depicting the manual scores for NOX4 RNA (0–4) on the TMA from 90 BC cases and 10 normal breast tissues. ISH scores were generated at × 200 magnification and recorded using the RNAscope system scoring guidelines: 0 = no staining; 1 = 1 to 3 dots per stroma cell; 2 = 4 to 10 dots per stroma cell; 3 = more than 10 dots per stroma cell with less than 10% of stroma cells with dot clusters; and 4 = more than 10 dots per stroma cell with more than 10% of stroma cells with dot clusters. * represents *p* < 0.001 vs normal stroma. (**I**) Boxplot showing varying levels of Nox4 RNA expression (Average # of brown spots detected per cell in the stroma region) in different grades of breast cancer. * represents *p* < 0.005 vs normal stroma

We have since demonstrated the clinical relevance of Nox4 in primary human breast CAFs. As shown in Fig. 4A, all patient-derived CAFs showed a significant upregulation of Nox4 mRNA expression compared to the normal fibroblasts (RMFs), in agreement with the Oncomine analysis shown in Fig. 3B. These CAFs were isolated from breast carcinoma tumors of varying molecular subtypes and clinical stages (I to III), implying that Nox4 upregulation is a common feature in breast CAFs and that activation of this gene persisted throughout various stages. Western blot analysis confirmed the overexpression of Nox4 in these CAFs (Fig. 4B). As expected, these CAFs also showed a heightened ROS production as revealed by increased H_2_O_2_ levels (Fig. 4C). Inhibiting Nox4 activity with 20 uM of GKT137831 reduced the ROS levels to near the levels seen in RMF. A higher level of GKT137831 (30 uM) further reduced the levels of the extracellular ROS in these CAFs (Fig. 4C).

To further determine the cellular redox status of CAFs, we assessed their glutathione (GSH) levels. GSH is a predominant small molecular antioxidant in cells. As GSH can be oxidized by ROS into GSSG, a reduction in the GSH:GSSG ratios as seen in CAFs (Fig. 4F) indicates an increase in cellular oxidative status. We have also observed an upregulation of p22 phox mRNA in CAFs (Additional file 2: Fig. S2). Since p22 phox is a critical component in superoxide-generating function of the Nox enzymes, our data suggest that activation of Nox4 in these CAFs is partly promoted by the upregulated p22 phox.

To further show the clinical significance of Nox4, we determined Nox4 transcription levels in a panel breast tumor microarray (TMA). Due to non-specificity issues related to most of the commercially available Nox4 antibodies for IHC staining, we chose to utilize RNA in situ* hybridization* (ISH) method, with the Nox4-specific RNAscope® assay. Figure 4G shows the Nox4-specific RNAsope staining in a panel of breast tumor tissues of various clinical grades. None to low levels of staining were detected in the stroma area of the normal tissues while significantly higher staining was observed in the tumor stroma. The RNAscope® scoring is tabulated and shown in Fig. 4H. The boxplot in Fig. 4I shows the average # of staining spots detected per stroma cell across the tumor grade. Significantly higher levels of Nox4 mRNA expression were seen in correlation with the tumor grade in the stroma region.

### Primary breast CAFs displays an activated phenotype

**Fig. 5:**
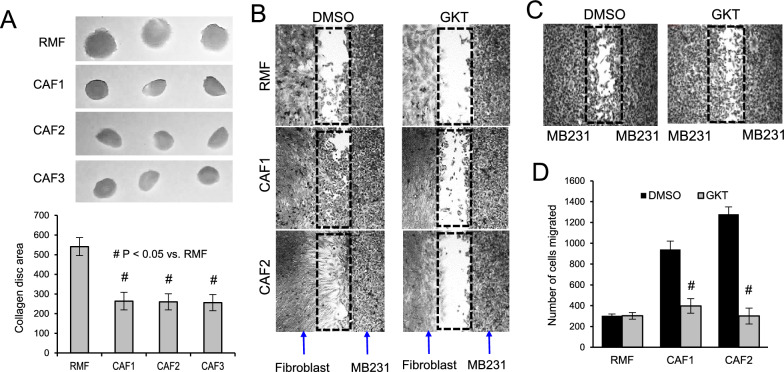
Activated phenotype of breast CAFs. (**A**) Fibroblasts were embedded in collagen matrix. Surface areas of the contracted collagen disc after 8 h were analyzed with ImageJ and presented in the lower bar graph. Pictures show representative triplicate of contracted collagen discs from *N* = 3 independent experiments. Data are mean ± SD of N = 3. (**B**) Migration of breast cancer cells when co-cultured with fibroblasts. The two cell types were seeded separately in ibidi culture inserts. When cells reached confluence, the inserts were removed to allow cells to migrate w/wo GKT137831 (20 μM). After 16 h of migration, stained cells were imaged for quantification by ImageJ, as shown in (**D**). (**C**) Migration of MDA-MB231 cells in mono-culture was not significantly affected by GKT137831. Representative images of *N* = 3 independent experiments are shown in (**B** and **C**). Data are mean ± SD of *N* = 3 independent experiments. # *p* < 0.05 in GKT-treated samples vs DMSO

To verify that the panel of our patient-derived CAFs show a more aggressive or activated myofibroblast-like phenotype, we first determined their ability to contract type I collagen, as described previously [4]. Figure 5A shows that CAF1-3 are more contractile (smaller collagen discs) compared to RMF. These CAFs are also more stimulatory in promoting cancer cell migration (Fig. 5B–D). The CAF-mediated cancer cell migration was significantly attenuated in the presence of GKT137831, while this Nox4 inhibitor had no effect on the migration of the cancer cells alone (Fig. 5C). These data indicate that these breast CAFs have an activated phenotype.

Since CAF5 shows the highest levels of Nox4 mRNA expression in the breast CAF panel, as shown in Fig. 3A, we also investigated the effect of GKT137831 on its phenotype. Additional file 3: Fig. S3 shows that GKT137831 significantly reduced the ability of CAF5 to contract collagen, to promote migration of breast cancer cells, and to generate ROS. These observations imply that there is an equivalent functionality of Nox4 in these CAFs regardless of differential expression levels of this pro-oxidative enzyme.

### Nox4 promotes an autophagic phenotype in CAFs

**Fig. 6:**
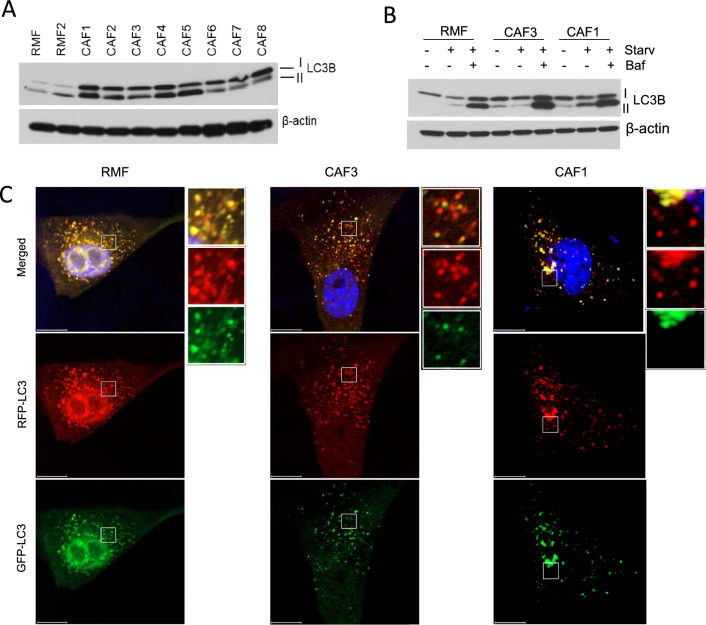
Autophagy phenotype in CAFs. (**A**) Western blot analysis showing increased levels of an autophagy marker, LC3B in all CAFs examined compared to the RMFs, under a basal culture condition. (**B**) Autophagy flux analysis. Fibroblasts were nutrient starved (HBSS buffer) for 6 h with or without treatment with the autophagy inhibitor bafilomycin A1 (Baf; 100 nM). Additional increased in LC3B expression in response to starvation and the lysosomal inhibitors were observed in CAFs. (**C**) Autophagic flux was further assessed using a tandem fluorescent-tagged ptfl-LC3 plasmid (mRFP-EGFP-LC3). Confocal microscopy analysis was performed after 24 h of transfection. At this time point, RMF still showed extensive acidified autophagosomal structures (yellow merged punta), while both CAFs showed a much reduced levels of GFP-LC3, as reflected by the remaining red puncta in the merged images. Representative GFP-LC3, RFP-LC3 and overlay images are shown. Right panels are enlarged photographs from the boxed areas. Scale bar represents 10 μm

Since ROS are linked to stress-induced autophagy, we next determined if Nox4-generated ROS plays a role in inducing autophagy. We found that levels of LC3B, an autophagy marker, are promoted under basal conditions in all CAFs as compared to RMFs (Fig. 6A). The observed increase in LC3-II levels could be attributed either to an increase in autophagic trafficking, or to a decrease in autophagic degradation, as LC3-II is degraded by the lysosome. Therefore, we performed autophagic flux assays in which a subset of cells was treated with bafilomycin A (Baf) to prevent autophagosome-lysosome fusion. The increase in autophagic flux was verified under starvation (HBSS buffer) where a further increase in LC3B levels were observed in CAFs in the presence of Baf (Fig. 6B). We also performed confocal microscopy analysis using a tandem repeat RFP-GFP-LC3 construct, as described [21]. Figure 6C shows a reduction in green fluorescent puncta at 24 h post-transfection in CAF1 and CAF3, resulting in an overall increase in red puncta on the merged confocal images. Meanwhile, the puncta in RMFs are mostly yellow in the merged image. These experiments suggest that CAFs have high basal autophagy, with further induction of autophagy observed under metabolic stress.

### Nox4 promotes autophagy-mediated survival in CAFs

**Fig. 7:**
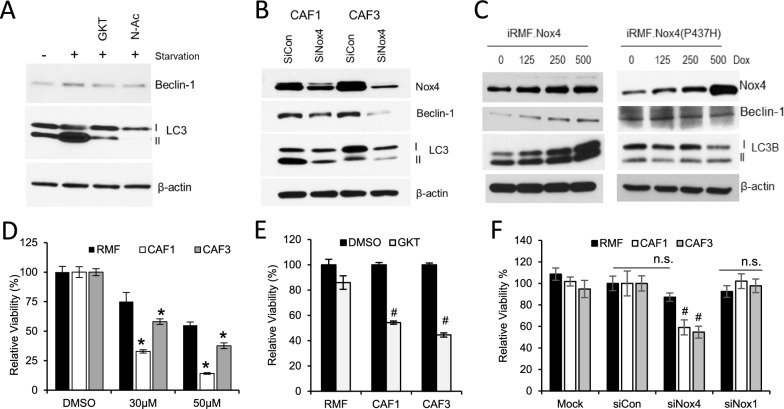
Nox4 promotes expression of the autophagy marker, LC3B in fibroblasts. (**A**) ROS-generating activity of Nox4 was targeted by treating CAF1 with a Nox4 inhibitor, GKT137831 (20 μM) or with a H_2_O_2_ scavenger, N-acetyl cysteine (N-Ac; 5 μM). After 24 h of treatment, protein lysate was collected and analyzed for the expression of the autophagy markers. (**B**) Nox4 expression was inhibited with a Nox4-specific siRNA in CAFs. After 24 h of siRNA transfection, CAFs were harvested for western blot analysis to evaluate the levels of beclin-1 and LC3B. (**C**) RMF were generated to express either a wild type Nox4 or an inactive mutant Nox4 (P437H) under doxycycline-mediated induction (Dox). The inducible RMF (iRMFs) were treated with indicated concentrations of Dox for 24 h prior to cell lysis for western blot analysis. Representative blots from *N* = 3 independent experiments are shown. (**D**) CAF viability was determined by PrestoBlue assay following 48 h of chloroquine (CQ) treatment. * represents p < 0.05 vs RMF treated at the same dosage of CQ. (**E**) GKT137831 (20 uM) reduced viability of CAFs compared to RMF. Fibroblast viability was measured by PrestoBlue reagent using a fluorescence plate reader after 5 days of GKT treatment. # represents *p* < 0.01 vs RMF. (**F**) Relative viability of fibroblasts after 48 h of siRNA-mediated knock down of Nox1 and Nox4. # represents p < 0.005 vs non-targeting siRNA (siCon) transfected CAFs and siNox4 transfected RMF. All error bars show SD of *N* = 3

To further show that Nox4 activation promotes autophagy, we inhibited Nox4 with GKT137831 or siNox4 and scavenged ROS with N-acetyl cysteine (NAC). Figure 7A shows that under starvation-induced autophagic conditions, inhibition of Nox4 activity downregulated beclin-1 (an autophagy initiating molecule that acts upstream of LC3) and LC3B levels. Suppression of Nox4 expression with siNox4 also downregulated expression levels of these autophagy markers in CAFs, as shown in Fig. 7B. We then asked if Nox4 expression alone can affect autophagy in normal mammary fibroblasts. Figure 7C shows that overexpression of the wild type Nox4, but not the inactive mutant, promoted the expression of autophagy markers in a dose dependent manner in RMFs. This mutation, P437H inhibits NADPH binding and hence the blocking the ability of Nox4 to generate ROS [22]. We have indeed confirmed that the P437H mutant Nox4 is defective in generating ROS (Additional file 4: Fig. S4). These data support a role of pro-oxidative Nox4 in promoting an autophagic phenotype in CAFs.

To determine if these CAFs rely on an autophagy process for survival, we treated them with chloroquine (CQ) which inhibits lysosomal acidification and, thus, blocks autolysosome formation, eventually leading to cell death. Figure 7D shows that CAFs are indeed more sensitive to CQ compared to RMFs, suggesting a dependency of CAFs on autophagic survival. In support of this, inhibition of Nox4 activity (Fig. 7E) and expression significantly reduced viability of these CAFs. Although being more selective for Nox4, GKT137831 can also target Nox1 activity due to a high similarity between Nox4 and Nox1 protein structures [23]. Knockdown of Nox1, however showed no significant changes in CAF viability (Fig. 7F), suggesting a Nox4-specific role in regulating survival in these fibroblasts.

### Nox4 upregulates Nrf2-mediated adaptive stress response in CAFs

**Fig. 8:**
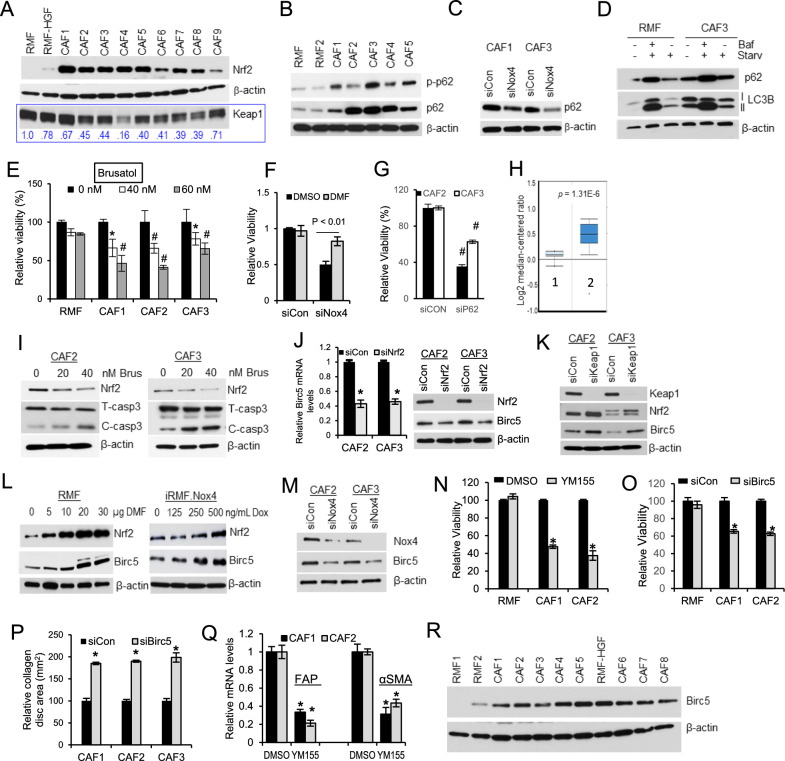
Nox4 promotes survival of CAFs via an up regulation of Nrf2 pathway. (**A**) Western blot analysis showing expression levels of Nrf2 and KEAP1 in total lysates of CAFs versus RMFs. Relative signal intensity of Keap1 (normalized to the actin control) was shown in numerical numbers. (**B**) Total and phosphorylated (Ser349) levels of p62 in CAFs. (**C**) Levels of p62 in CAFs after 24 h of siNox4 transfection. (D) Starvation induced autophagy resulted in an increase in p62 accumulation in CAFs versus RMF. Fibroblasts were nutrient starved (HBSS buffer) for 6 h with or without treatment with the autophagy inhibitor, bafilomycin A1 (Baf; 100 nM). (**E**)Targeting Nrf2 impaired viability of CAFs compared to RMF. Confluent fibroblasts were treated with varying concentrations of brusatol and viability of cells were determined after 48 h of treatment. * represents *p* < 0.05 vs untreated CAFs, # represents *p* < 0.01 vs untreated CAFs. (**F**) Survival of CAFs after 48 h of siNox4 transfection in the presence or absence of DMF (30 uM). (**G**) Viability of CAFs after 48 h of sip62 transfection. # represents *p* < 0.01 vs untreated CAFs. (**H**) Oncomine microarray analysis of p62 mRNA expression in breast stroma vs normal stroma in the Finak et al. dataset. 1: Normal stroma, *N* = 6; 2: Tumor stroma, *N* = 53. (I) Inhibition of Nrf2 with brusatol induced caspase-3 activation in CAFs. T-Casp3 = total caspase-3; C-casp3 = cleaved caspase-3. (J) Bar graph—Relative mRNA expression of Birc5 in CAFs after 48 h of siNrf2 transfection. *N* = 3, * represents *p* < 0.05 vs siCon transfected CAFs. Right panel -Western blot analysis of Birc5 expression after 48 h of siNrf2 transfection. (**K**) Suppressing the expression of Keap1, a negative regulator of Nrf2, also resulted in an induction of Birc5 protein expression in CAFs. (**L**) Chemical induction of Nrf2 with DMF (ug) in RMF (left panel) or upstream overexpression of Nox4 with doxycycline (ng/mL) in the inducible RMF (iRMF.Nox4) (right panel) both increased the expression levels of Birc5. (**M**) Knocking down expression of Nox4 in CAFs inhibited expression of Birc5 in CAFs. Viability of CAFs when Birc5 was targeted either with 200 nM of YM155 treatment (N) or with siBirc5 transfection (**O**), for 48 h. *N* = 3, * represents *p* < 0.05 vs DMSO treated CAFs. (**P**) Collagen contraction activity of CAFs after 48 h of siBirc5 transfection. (**Q**) RT-PCR analysis of CAF markers, FAP and αSMA after 48 h of YM155 treatment. (**R**) Western blot analysis of Birc5 expression in primary breast CAFs vs. RMFs. All Western blots are representative of *n* = 3 separate experiments. Error bars are SD of *n* = 3

Next, we determined if these breast CAFs have adapted to cope with the Nox4-induced oxidative stress. One such adaptive mechanisms that we have investigated in these CAFs is the Nrf2 pathway, which is considered a master regulator of cellular stress against oxidative insults [15]. Indeed, we observed increased protein levels of Nrf2 expression of the panel of breast CAFs vs. the RMF (Fig. 8A). We have also observed that these CAFs are more resistant to exogenous ROS insults when compared to the RMF (Additional file 5: Fig. S5). Figure 8A also shows downregulation of the major Nrf2 negative regulator, Keap1 in the majority of these CAFs. We then evaluated the level of p62, which has been reported to affect Nrf2 accumulation [24, 25]. As shown in Fig. 8B, CAFs showed a concomitant increase in p62 levels (both total and S349 phosphorylated proteins), compared to RMF. Inhibition of Nox4 expression with siNox4 resulted in a reduction of p62 levels in CAFs (Fig. 8C). We have further shown that upon starvation-induced autophagy activation, there is an increased accumulation of p62 levels in CAFs when compared to the RMF (Fig. 8D). Our data suggest that the increased expression of Nrf2 in CAFs is likely due to p62-mediated autophagic degradation of Keap1. Although p62 is itself a substrate of autophagy degradation, these sustained levels of p62 seen in CAFs cannot be explained by a defective autophagy but the accumulation of p62 is most likely due to an increase in its transcriptional regulation by Nrf2, since there is an antioxidant response element (ARE) region present in the promoter of p62, as discussed [26]. This allows for a positive feed-forward Nrf2-p62 signaling.

To further show a functional consequence of Nrf2 activation in CAFs, we chemically inhibited Nrf2 with brusatol [27] and found that CAFs are more sensitive to brusatol compared to RMFs (Fig. 8E). More importantly, we have demonstrated that Nox4-mediated survival is dependent on Nrf2 response in CAFs. Figure 8F shows that dimethyl fumarate (DMF), an inducer of Nrf2 [28] prevented siNox4-mediated loss of viability in CAFs. When p62 expression was downregulated, CAF viability was also suppressed (Fig. 8G). In addition, an Oncomine analysis reveals a significant upregulation of p62 gene expression in breast tumor stroma vs. normal stroma as shown in Fig. 8H [29]. This suggests a clinical significance of p62 upregulation in breast CAFs.

Furthermore, we have shown that Nrf2 promoted survival of CAFs by inhibiting caspase-3 cleavage. Figure 8I shows that inactivating Nrf2 in CAFs with brusatol treatment resulted in an increase in the protein levels of cleaved caspase-3. This pro-survival function is likely mediated via Nrf2-induced Birc5 (survivin) expression. Birc5 (Baculoviral IAP Repeat Containing 5, also known as survivin) acts at the crossroads of multiple cancer pathways, which include regulations of mitosis and apoptosis [30]. As shown in Fig. 8J, downregulation of Nrf2 expression with siNrf2 resulted in reduction of Birc5 mRNA levels (left panel) and protein expression (right panel). (K) Conversely, promotion of Nrf2 expression by suppressing the expression of its negative regulator, Keap1 with siKeap1, significantly induced expression of Birc5 in CAFs. Similarly, activation of Nrf2 either chemically with DMF or genetically in a dox-inducible normal fibroblasts (iRMF.Nox4), increased the expression levels of Birc5 (Fig. 8L). Figure 8M shows that knocking down expression of Nox4 with siNox4 also inhibited expression of Birc5 in CAFs.

We next demonstrated that Birc5 regulates a survival phenotype of CAFs. Targeting Birc5 with 200 nM of YM155 (Fig. 8N) and with siBirc5 (Fig. 8O) significantly attenuated the viability of CAFs. To further demonstrate the effect of Birc5 on CAF phenotype, we determined their collagen contraction activity after siBirc5 transfection. Figure 8P shows that siBirc5 significantly suppressed their ability to contract collagen, resulting in larger collagen disc areas, as compared to siCon transfection. We then asked if Birc5 regulates activation of CAFs. As shown in Fig. 8Q, inhibition of Birc5 with YM155 significantly decreased the mRNA expression of two CAF markers, FAP and αSMA. Furthermore, we have shown that expression levels of the pro-survival factor, Birc5 are upregulated in a panel of primary breast CAFs when compared to RMFs (Fig. 8R). Altogether, our data demonstrate that Nox4 activates the Nrf2 adaptive stress response that promotes a pro-survival mechanism in CAFs, partly via induction of Birc5.

## Discussion

It is well recognized that an oxidative tumor microenvironment (TME) contributes to tumor progression, metastasis, recurrence, and therapeutic resistance [31]. While ROS or oxidative stress can originate from various sources in neoplastic epithelial cells, including from altered metabolism, oncogene expression, and heightened proliferative signals, there is a gap in our understanding of the role played by the stromal components in these phenomena. Our study here points to CAFs as one of the major contributors to the oxidative TME, via upregulation of Nox4. Hanley et al. [32] have suggested Nox4 as a potentially promising CAF target based on Nox4 and αSMA IHC staining in stromal regions of human samples (head and neck squamous cell carcinoma, esophageal adenocarcinoma, and colon adenocarcinoma), as well as in vivo findings using TGFβ-activated skin fibroblasts. Increased Nox4 gene expression has also been reported in prostate cancer-associated stroma [33]. While these small number of studies support a tumor-promoting role of Nox4 derived from activated fibroblasts, direct evidence linking this pro-oxidant to the tumor-supporting CAF phenotype and the mechanisms involved is lacking, particularly in breast cancer. This report, by utilizing a panel of patient-derived breast CAFs, in addition to an experimental-CAF model [4] (RMF-HGF) support a role of Nox4 in inducing an autophagic CAF phenotype and a pro-survival Nrf2-Birc5 stress response, thereby promoting mammary tumorigenesis and metastasis.

High Nox4 activation can be detrimental to cells during severe stress. An adaptive antioxidant mechanism in counteracting this chronically high Nox4 context is likely involved to maintain an optimum range of ROS for CAF survival. Indeed, we observed the levels of the master stress regulator, Nrf2 are upregulated in all CAFs (Fig. 6). Nrf2 is a transcription factor that is activated in response to oxidative stress and electrophilic stress. Hyperactivation of Nrf2 in tumors creates an environment that favor the survival of cancer cells by protecting them from excessive oxidative stress, chemotherapeutic agents, or radiotherapy. Upregulation of Nrf2 is therefore, strongly associated with poor patient prognosis and therapeutic resistance [17]. While much is known about the role of Nrf2 in cancer cells, very little is known regarding the implication of this adaptive response pathway in CAFs. Although a proteomic analysis showed upregulation of some Nrf2-targeted proteins in chemo-induced activated fibroblasts [34] and CXCL14-induced activated fibroblasts [35], demonstrating that hyperactivation of Nrf2 occurs in breast CAFs has not been reported.

Cancer cells rely on autophagic flux to promote growth and survival [36]. This has led to numerous clinical trials focusing on inhibiting autophagy in cancer. Chloroquine and hydrochloroquine (CQ and HCQ) are currently the only clinically available drugs that target autophagy [37]. Despite promising outcomes, further investigation is needed to elucidate the molecular mechanisms of these compounds not just in cancer cells but in other non-cancerous cell types in the TME. The dependency of CAFs on autophagy is not well established. Heightened basal autophagy has been reported in stroma fibroblasts of head and neck cancers [38] and prostate cancer [39]. However, the molecular mechanism underlying these phenomena is not well defined. In another study, normal fibroblasts that were co-cultured with breast cancer cells showed an autophagic phenotype [40), but these studies did not investigate the significance of autophagy in patient-derived CAFs. It is not surprising that CAFs rely on autophagy-mediated pro-survival mechanisms as these fibroblasts are under tremendous metabolic demands and the addition of autophagy allows for a tolerance to higher energy stress while increasing bioavailability of biosynthesis materials. A few reports have shown an increase in autophagy in fibroblasts (mouse MEF or skin fibroblasts) when exposed to cancer cells [41, 42], suggesting that this process is not only exploited by cancer cells but also frequently adopted by stroma fibroblasts.

Sequestosome 1 (A.K.A. p62) has been shown to be another factor that can activate Nrf2 by directly binding to Keap1 and targeting Keap1 for selective autophagic degradation [24, 25]. It is overexpressed in many types of human cancers including breast cancer [43, 44] and its expression correlates with poor prognosis in patients with triple-negative breast cancer [45]. Further supporting a role of p62 in oncogenesis and as a potential CAF target, a p62-encoding DNA vaccine exhibited strong antitumor and anti-metastasis activity in four mouse tumor models, including mammary carcinoma [46]. This observation is in contrast to some studies reporting a down regulation of p62 expression in prostate tumor-associated stroma [47], experimentally activated hepatic stellate cells [48] and skin CAFs [49]. In this study, we clearly observed an increase in the levels of p62 protein expression (both total and S351-phosphorylated p62) in patient-derived CAFs. This S351-phosphorylation site was shown to be critical in regulating selective autophagy and Nrf2 activation [50]. Furthermore, Oncomine analysis also showed a significant upregulation of p62 gene expression in breast tumor stroma vs. normal stroma as shown in Fig. 8G [29] as well as in another breast tumor stroma microarray analysis [51]. In addition, Kang et al.[52] have recently showed that the p62-Nrf2 axis contributes to fibroblast activation and tumor progression of lung cancer. Together, these contradictory observations likely reflect the CAF heterogeneity and imply a context-dependent function of p62 in CAFs.

Although Nrf2 is well known to be a promising anti-cancer target, no FDA-approved drugs targeting Nrf2 activity in cancer have been realized to date [17]. The plethora of downstream targets (more than 100 identified so far) of this master regulator of cellular stress response also complicates the specific use of Nrf2-targeting approaches for cancers. Our data presented here suggest that Birc5 is a key downstream modulator of Nrf2 that promotes CAF phenotype and their survival. This is not surprising, as Birc5 has recently been shown to prevent excessive autophagy as a pro-survival mechanism in breast cancer [53, 54]. Moreover, Birc5 is overexpressed in aggressive cancers [55] where its presence correlates with increased resistance to chemotherapy [56] and irradiation [57]. We suspect that Nrf2 directly participates in transactivating gene expression of Birc5 in CAFs. This is based on an analysis of transcription binding motifs using the Eukaryote Promoter Database (JASPAR Core matrix profile MA0150.1). There are 2 predicted Nrf2 binding sites in the − 2 K upstream of TSS. Importantly, these sites also overlap with the MafG binding motifs (− 1227 and -414). Nrf2 does not act alone but can form heterodimers with MafG to co-occupy functional Nrf2 binding sites to participate in the transcriptional activation [58]. Further supporting this, in a lung tissue transcriptome analysis, *Birc5* was found to be one of the genes downregulated in Nrf2-/- mice when exposed to cigarette smoke [59]. The direct involvement of Nrf2 on Birc5 transcription was, however, not confirmed in this study. Additional studies will be needed in establishing the Nox4-Nrf2-Birc5 axis as a mediator of CAF survival to provide the rationale to exploit this redox vulnerability of CAFs.

There is a recent report showing that tissue-specific deletion of Nox4 gene promoted tumor formation in carcinogen-induced colorectal cancers and fibrosarcomas (61). The authors demonstrated that Nox4-generated ROS are critical for inducing DNA damage response upon exposure to carcinogens and that depleting Nox4 promoted genetic instability and tumor initiation. This study further highlights the biphasic effects of ROS and their differential roles during various stages of tumor initiation, malignant transformation, and progression. Based on the fact that multiple studies have shown the anti-tumor effect of Nox4 targeting approaches in established tumors (62,63), including breast cancers (64,65), in addition to the evidence presented in the present study, we strongly believe that Nox4 remains a promising CAF target for solid tumors and its role in breast cancer warrants more studies.

## Conclusions

In summary, our study supports a clinically relevant role of Nox4 in breast CAFs. Future focus on revealing the molecular basis of Nox4-mediated CAF phenotype will be critical in providing insight into this redox signaling mechanisms and potential new approaches targeted at their dependency on the autophagy or the Nrf2 response. The emerging insights into downstream regulators of Nrf2 signaling in CAFs will provide new opportunities for clinical interventions. This is particularly important as Birc5-targeting compounds such as YM155 have advanced into numerous different human cancer clinical trials. Although the clinical outcomes of these trials remain inconclusive, enthusiasm in targeting Birc5 for cancers remains high. The impact of CAFs on cancer progression is not limited to their direct influence on primary tumors, but also extends to other cellular components of the metastatic lesions. Therefore, modification of CAFs is expected to provide a promising therapeutic response, especially if applied in conjunction with other anti-cancer compounds such as chemotherapeutic agents.

### Supplementary Information

**Additional file1**: **Fig. S1.** Nox4-derived ROS does not contribute to macrophage phenotype. (**A**) Primary human monocytes were treated with an antioxidant, MnTE or GKT137831 during differentiation and polarization to M1 or M2 macrophages and analyzed for the mRNA expression of M2 markers, IL-10. (**B**) GKT137831-treated macrophages were also analyzed for extracellular release of ROS by AmplexRed assay. * represent *P* < 0.05 vs untreated control. Error bars are standard deviation of *N* =3.

**Additional file2**: **Fig S2.** Real time PCR analysis showing relative mRNA expression of (**A**) p22phox in CAFs vs RMF. * represent *P* < 0.05 vs RMF, *N* = 3. Error bars are standard deviation of *N* =3.

**Additional file3**: **Fig S3.** Activated phenotype of CAF5. (**A** and **B**) Fibroblasts were embedded in collagen matrix. Surface areas of the contracted collagen disc after 8 h were analyzed with ImageJ and presented in the bar graph. Pictures show representative triplicate of contracted collagen discs from *N* = 3 independent experiments. Data are mean ± SD of N = 3. * *p *< 0.05 in GKT treated samples versus DMSO. # p < 0.05 vs untreated CAF5. (**C**) Migration of breast cancer cells when co-cultured with CAF5. The two cell types were seeded separately in ibidi culture inserts. When cells reached confluence, the inserts were removed to allow cells to migrate w/wo GKT137831 (20 μM). After 16 h of migration, stained of migration, cells were imaged for quantification by ImageJ, as shown in (**D**). Data are mean ± SD of *N* = 3 independent experiments. * p < 0.05 in CAF5 versus RMF. # *p* < 0.05 in GKT treated CAF5 vs untreated CAF5. (**E**) GKT137831 effectively reduced cellular ROS levels in CAF5, as demonstrated by the levels of H2O2 released into the cell culture media via AmplexRed assay. * *p *< 0.05 vs untreated RMF. # *p *< 0.05 in GKT treated CAF5 vs untreated CAF5. Data are mean ± SD of *N *= 3 independent experiments.

**Additional file4**: **Fig S4.** Extracellular H2O2 levels in Nox4 overexpressing RMF. Nox4 expression (both wild type and the inactive mutant form) was induced with 100 ng/mL of doxycycline for 24 h and 48 h prior to AmplexRed assay. Extracellular H2O2 was measured after 30 min of reagent incubation. Error bars are standard deviations of mean from 3 separate samples. * *p* < 0.01 versus RMF at 48h.

**Additional file5**: **Fig. S5.** CAFs are more tolerant to exogenous ROS insults compared to RMF. Confluent fibroblasts were treated with H2O2 at the indicated concentrations and viability was assessed by PrestoBlue reagent after 4, 8, and 16 hours of treatment. * represents P < 0.05 vs RMF and # represents *p *< 0.005 versus RMF, treated at the same dose of H_2_O_2_.

## Data Availability

All data in our study are available upon request.

## Abbreviations

CAFs: Cancer-associated fibroblasts
Nox4: NADPH oxidase 4
Nox1: NADPH oxidase 1
ROS: Reactive oxygen species
Nrf2: Nuclear factor-erythroid factor 2-related factor 2
O_2_^•^^−^: Superoxide
H_2_O_2_: Hydrogen peroxide
RMF: Normal mammary fibroblasts isolated form reduction mammoplasty
RMF-HGF: RMF generated to overexpress hepatocyte growth factor
SDF1: Stroma-derived factor 1
αSMA: Alpha smooth muscle actin
MnTE: Manganese porphyrin, MnTE-2-PyP
PDGFRα: Platelet-derived growth factor receptor alpha
GSH: Glutathione
GSSG: Oxidized glutathione
Dox: Doxycycline
Baf: Bafilomycin
DMF: Dimethyl fumarate
Birc5: Baculoviral IAP Repeat Containing 5, a.k.a. survivin
