# Supplementary figures and images for: Upregulation of Nox4 induces a pro-survival Nrf2 response in cancer-associated fibroblasts that promotes tumorigenesis and metastasis, in part via Birc5 induction
Source: Breast Cancer Res. 2022 Jul 14;24:48. doi: 10.1186/s13058-022-01548-6 (PMC9281082; doi:10.1186/s13058-022-01548-6)

## Slide 1
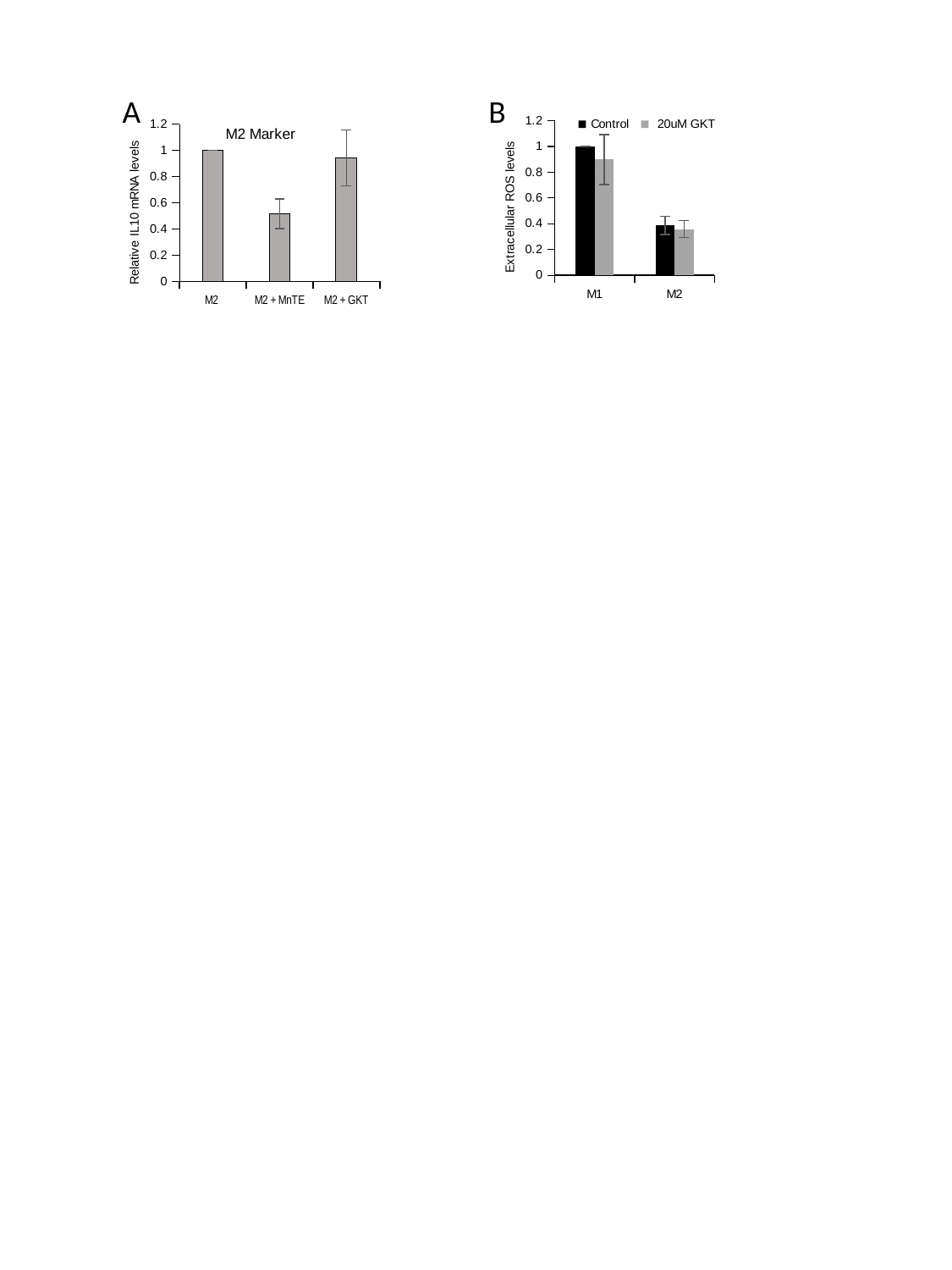

A
B
### Chart
| Category | Control | 20uM GKT |
|---|---|---|
| M1 | 1.0 | 0.8984843789621014 |
| M2 | 0.38706759380044187 | 0.35899773437294485 |
### Chart: M2 Marker
| Category | |
|---|---|
| M2 | 1.0 |
| M2 + MnTE | 0.5163286502416222 |
| M2 + GKT | 0.9427845359182377 |

Supplement: Supplementary file 1 — Additional file1: Fig. S1. Nox4-derived ROS does not contribute to macrophage phenotype. (A) Primary human monocytes were treated with an antioxidant, MnTE or GKT137831 during differentiation and polarization to M1 or M2 macrophages and analyzed for the mRNA expression of M2 markers, IL-10. (B) GKT137831-treated macrophages were also analyzed for extracellular release of ROS by AmplexRed assay. * represent P < 0.05 vs untreated control. Error bars are standard deviation of N =3. [file 13058_2022_1548_MOESM1_ESM.pptx]

## Slide 1
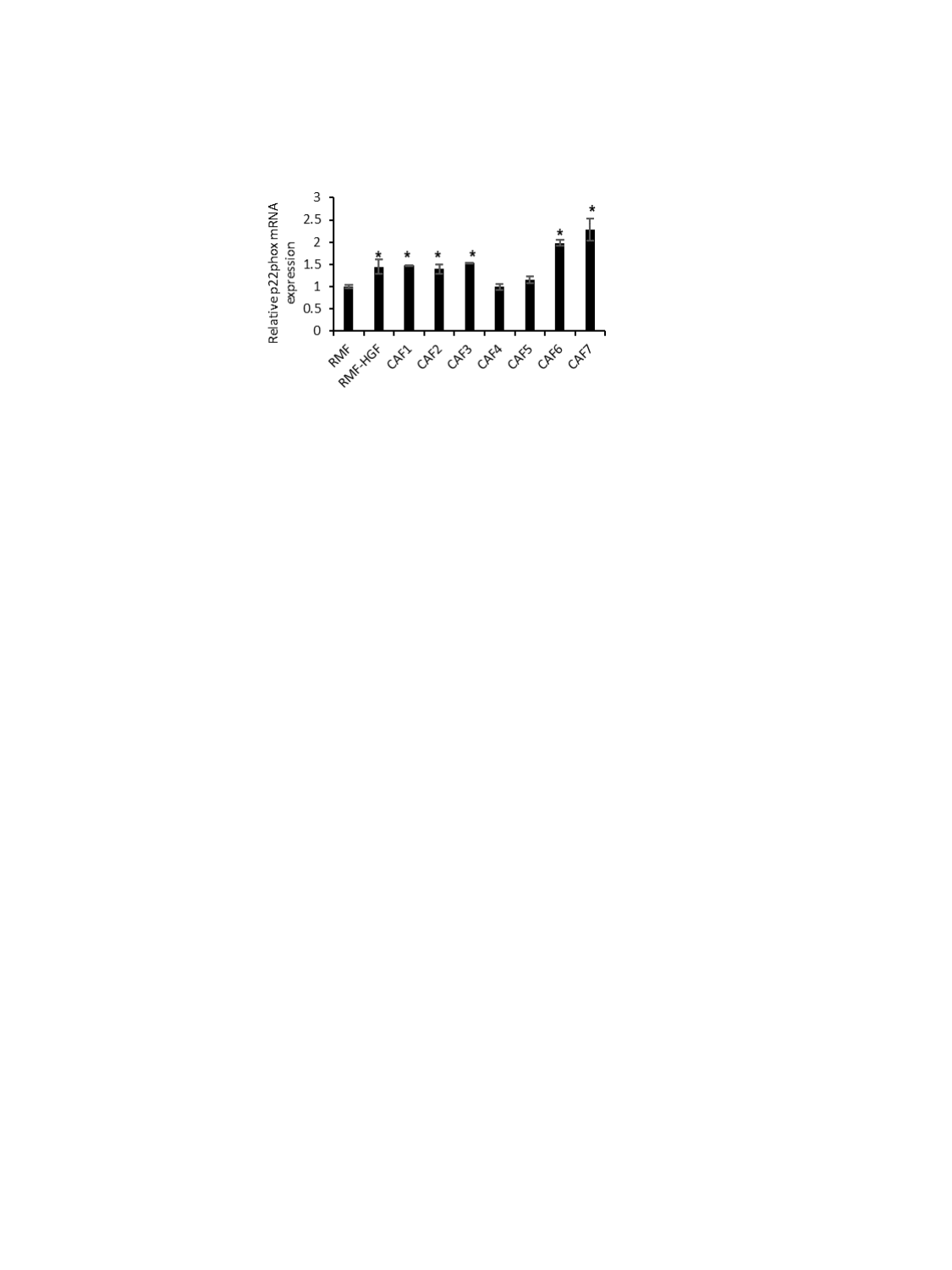

Supplement: Supplementary file 2 — Additional file2: Fig S2. Real time PCR analysis showing relative mRNA expression of (A) p22phox in CAFs vs RMF. * represent P < 0.05 vs RMF, N = 3. Error bars are standard deviation of N =3. [file 13058_2022_1548_MOESM2_ESM.pptx]

## Slide 1
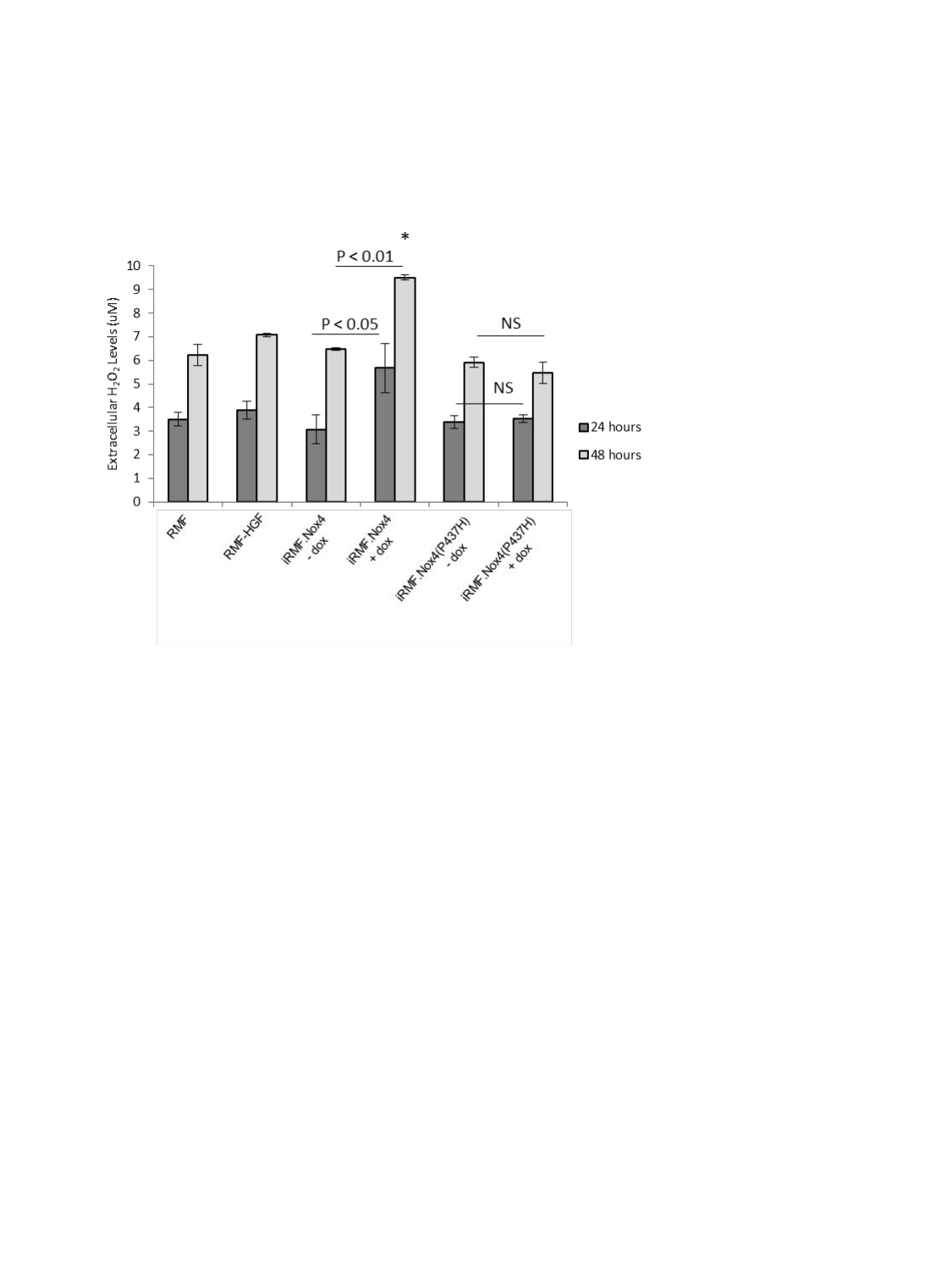

Supplement: Supplementary file 4 — Additional file4: Fig S4. Extracellular H2O2 levels in Nox4 overexpressing RMF. Nox4 expression (both wild type and the inactive mutant form) was induced with 100 ng/mL of doxycycline for 24 h and 48 h prior to AmplexRed assay. Extracellular H2O2 was measured after 30 min of reagent incubation. Error bars are standard deviations of mean from 3 separate samples. * p < 0.01 versus RMF at 48h. [file 13058_2022_1548_MOESM4_ESM.pptx]

## Slide 1
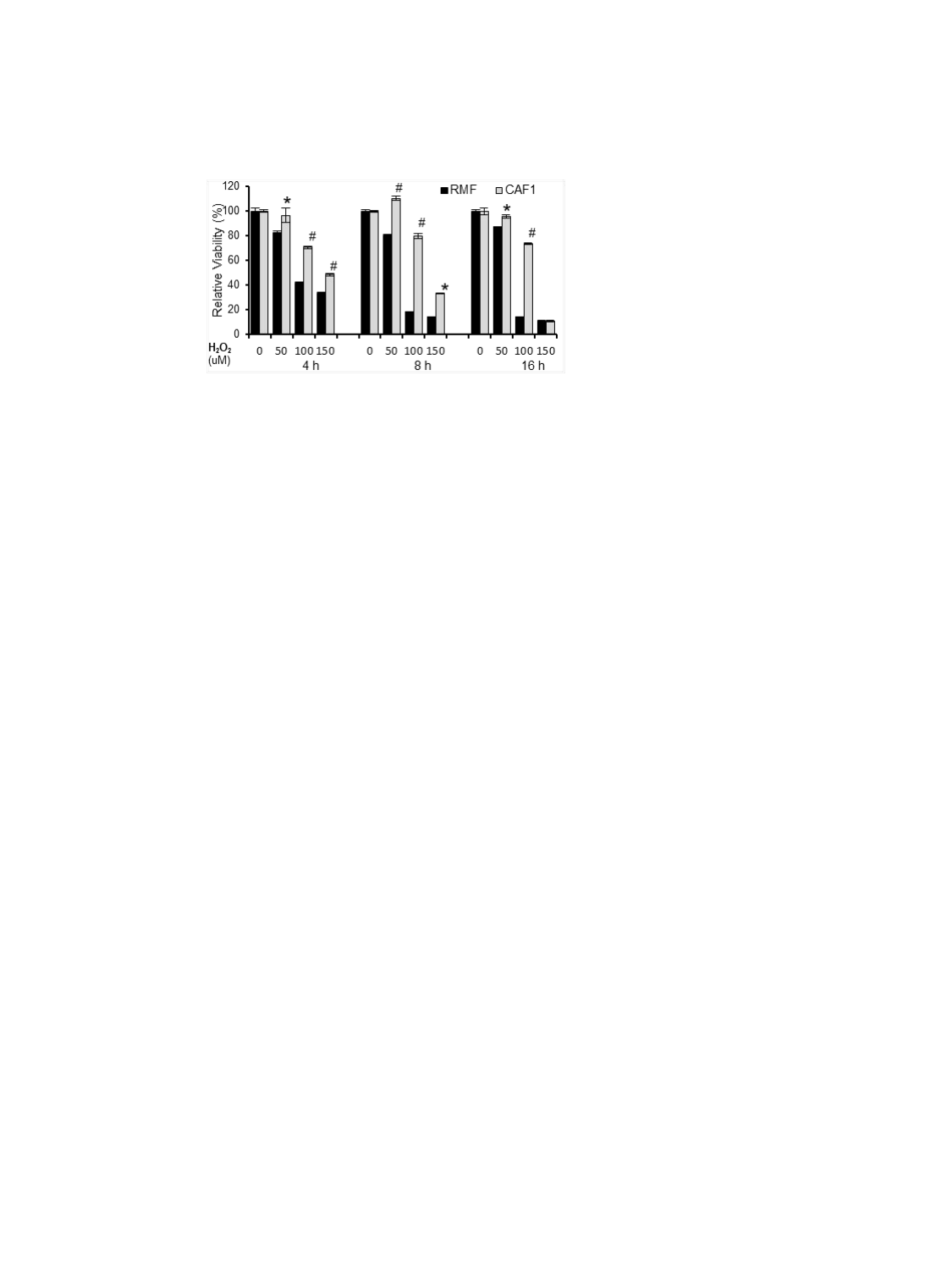

Supplement: Supplementary file 5 — Additional file5: Fig. S5. CAFs are more tolerant to exogenous ROS insults compared to RMF. Confluent fibroblasts were treated with H2O2 at the indicated concentrations and viability was assessed by PrestoBlue reagent after 4, 8, and 16 hours of treatment. * represents P < 0.05 vs RMF and # represents p < 0.005 versus RMF, treated at the same dose of H2O2. [file 13058_2022_1548_MOESM5_ESM.pptx]
