# Supplementary material for: Upregulation of Nox4 induces a pro-survival Nrf2 response in cancer-associated fibroblasts that promotes tumorigenesis and metastasis, in part via Birc5 induction
Source: Breast Cancer Res. 2022 Jul 14;24:48. doi: 10.1186/s13058-022-01548-6 (PMC9281082; doi:10.1186/s13058-022-01548-6)
Supplement: Supplementary file 3 — Additional file3: Fig S3. Activated phenotype of CAF5. (A and B) Fibroblasts were embedded in collagen matrix. Surface areas of the contracted collagen disc after 8 h were analyzed with ImageJ and presented in the bar graph. Pictures show representative triplicate of contracted collagen discs from N = 3 independent experiments. Data are mean ± SD of N = 3. * p < 0.05 in GKT treated samples versus DMSO. # p < 0.05 vs untreated CAF5. (C) Migration of breast cancer cells when co-cultured with CAF5. The two cell types were seeded separately in ibidi culture inserts. When cells reached confluence, the inserts were removed to allow cells to migrate w/wo GKT137831 (20 μM). After 16 h of migration, stained of migration, cells were imaged for quantification by ImageJ, as shown in (D). Data are mean ± SD of N = 3 independent experiments. * p < 0.05 in CAF5 versus RMF. # p < 0.05 in GKT treated CAF5 vs untreated CAF5. (E) GKT137831 effectively reduced cellular ROS levels in CAF5, as demonstrated by the levels of H2O2 released into the cell culture media via AmplexRed assay. * p < 0.05 vs untreated RMF. # p < 0.05 in GKT treated CAF5 vs untreated CAF5. Data are mean ± SD of N = 3 independent experiments. [file 13058_2022_1548_MOESM3_ESM.pptx]

## Slide 1
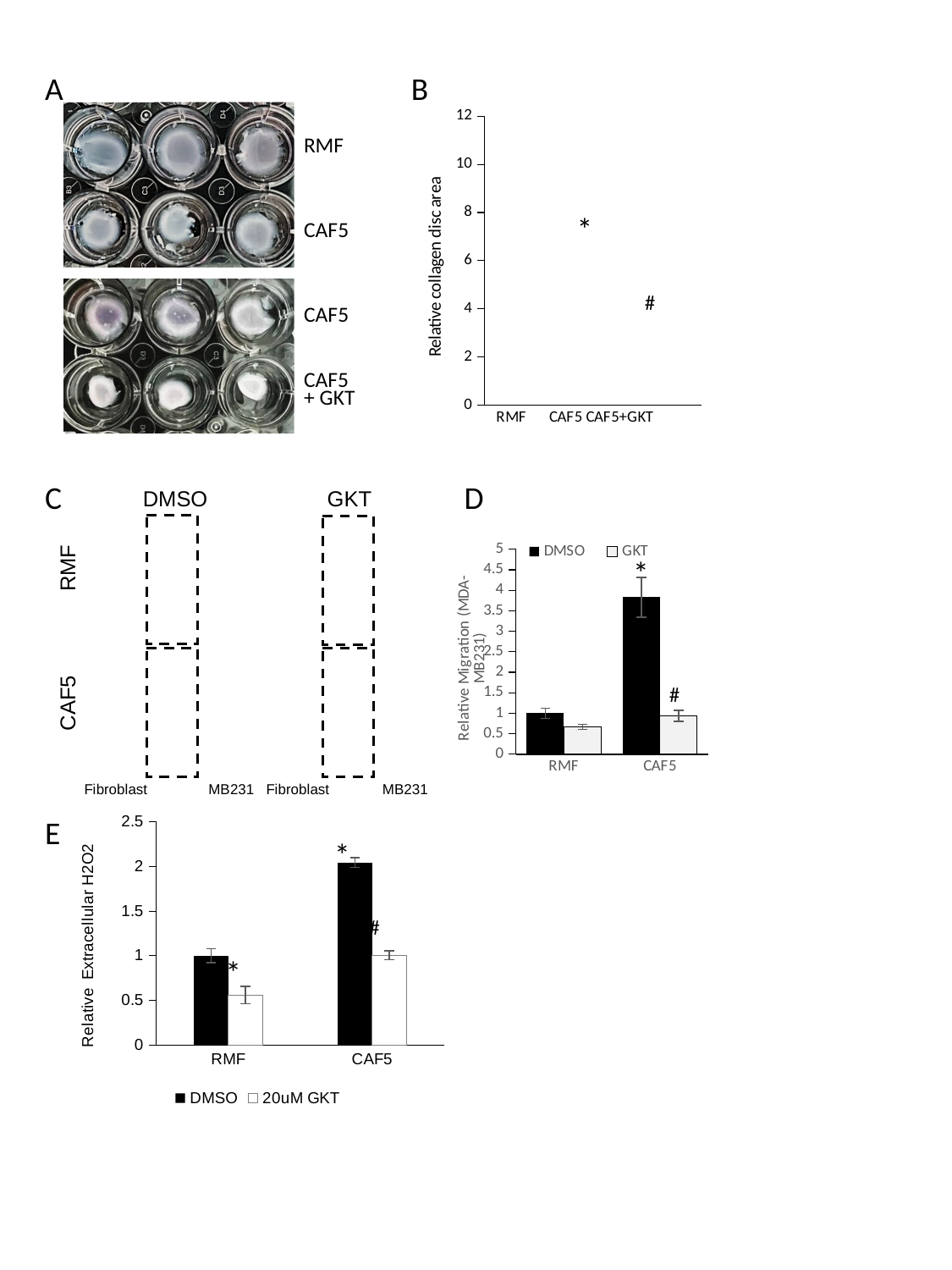

A
B
### Chart
| Category | |
|---|---|
| RMF | 0.9999999999999999 |
| CAF5 | 0.6191597716288202 |
| CAF5+GKT | 0.27365192132373733 |
*
#
C
D
### Chart
| Category | DMSO | GKT |
|---|---|---|
| RMF | 1.0 | 0.67 |
| CAF5 | 3.83 | 0.935 |*
#
E
### Chart
| Category | DMSO | 20uM GKT |
|---|---|---|
| RMF | 1.0 | 0.56 |
| CAF5 | 2.0447634687715213 | 1.0065158658685172 |*
#
*
